# TWENTY-FOUR-HOUR ACTIVITY COMPOSITION ASSOCIATIONS WITH MUSCLE POWER AND STRENGTH IN OLDER MEN AND WOMEN

**DOI:** 10.1093/geroni/igae098.2406

**Published:** 2024-12-31

**Authors:** Lauren Roe, Elsa Strotmeyer, Nancy Glynn, Peggy Cawthon, Yan Ma, Megan Hetherington-Rauth, Stephen Kritchevsky, Jane Cauley

**Affiliations:** University of Pittsburgh School of Public Health, Sewickley, Pennsylvania, United States; University of Pittsburgh, Pittsburgh, Pennsylvania, United States; University of Pittsburgh School of Public Health, Pittsburgh, Pennsylvania, United States; California Pacific Medical Center, San Francisco, California, United States; University of Pittsburgh School of Public Health, Pittsburgh, Pennsylvania, United States; California Pacific Medical Center, San Francisco, California, United States; Wake Forest University School of Medicine, Wake Forest University School of Medicine, North Carolina, United States; University of Pittsburgh School of Public Health, Pittsburgh, Pennsylvania, United States

## Abstract

Physical activity (PA) and sedentary (SED) behavior have opposing associations with skeletal muscle function and are often studied separately, but are codependent. We evaluated associations of 24-hr movement behaviors [moderate-to-vigorous intensity (MVPA), light intensity (LPA), SED, and sleep] with muscle power and strength in 714 community-dwelling older adults (58% women, 76±5 years) from the Study of Muscle, Mobility and Aging (SOMMA). Compositional data analysis applied to 24-hr accelerometry (wrist-worn) determined proportions of time spent in each behavior relative to the remaining behaviors. Multiple linear regression evaluated associations between 24-hr composition with peak stair climb ascend power and leg press power (W), and maximum leg press strength and grip strength (kg). Compositional isotemporal substitution methods determined theoretical changes in the outcome if 10-60 minutes of one behavior was displaced by another in 10-minute increments. Higher proportions of MVPA vs. other behaviors were associated with higher leg press power in men (β: 67.5, 95% CI: 19.9, 115.1) and women (β: 29.1, 95% CI: 8.1, 50.2), and higher stair climb power in women (β: 25.0, 95% CI: 16.5, 33.4). Higher proportions of LPA vs. remaining behaviors were associated with lower leg press power in men (β: -117.9, 95% CI: -193.7, -42.1) and women (β: -39.0, 95% CI: -73.5, -4.6), and lower stair climb power in women (β: -31.6, 95% CI: -45.5, -17.8). No significant associations were found for strength. The most effective time-use substitution to improve muscle power may be through displacing SED or LPA with MVPA in older men and women.

